# The Associations between Bridal Pregnancy and Obstetric Outcomes among Live Births in Korea: Population-Based Study

**DOI:** 10.1371/journal.pone.0103178

**Published:** 2014-08-08

**Authors:** Jung-Yun Lee, Joong Shin Park, Jong Kwan Jun, Seung Han Shin, Young-Jin Ko, Sang Min Park

**Affiliations:** 1 Department of Obstetrics and Gynecology, Seoul National University College of Medicine, Seoul, Korea; 2 Department of Pediatrics, Seoul National University College of Medicine, Seoul, Korea; 3 Department of Family Medicine, Seoul National University College of Medicine, Seoul, Korea; Penang Medical College, Malaysia

## Abstract

**Objective:**

In East Asia the recently increased number of marriages in response to pregnancy is an important social issue. This study evaluated the association of marriage preceded by pregnancy (bridal pregnancy) with obstetric outcomes among live births in Korea.

**Methods:**

In this population-based study, 1,152,593 first singleton births were evaluated from data registered in the national birth registration database from 2004 to 2008 in Korea. In the study population, the pregnancy outcomes among live births from the bridal pregnancy group (N = 62,590) were compared with the outcomes of the post-marital pregnancy group (N = 564,749), composed of women who gave birth after 10 months but before 24 months of marriage. The variables preterm birth (PTB; <37 weeks gestation) and low birth weight (LBW; <2.5 kg) were used to determine the primary outcome. The adjusted odds ratios (aORs) and 95% confidence intervals (CIs) were calculated after controlling for socio-demographic factors.

**Results:**

The socio-demographic factors among the bridal pregnancy group were associated with a social disadvantage and particular risk factors. In the subgroup analyses of maternal age, differences in adverse pregnancy outcomes from bridal pregnancy were identified between women in the following age group: (i) ≤19, (ii) 20–39, and (iii) ≥40 years. After the multivariate analysis, the aORs for each age group were 1.47 (95% CI: 1.15–1.89), 1.76 (1.70–1.83), and 1.13 (0.77–1.66), respectively, for PTB and 0.92 (0.70–1.21), 1.60 (1.53–1.66), and 1.11 (0.71–1.74), respectively, for LBW. In the adjusted logistic regression models, bridal pregnancy was associated with PTB (1.76, 1.69–1.82) and LBW (1.53, 1.48–1.59).

**Conclusion:**

Pregnancy outcomes among live births from bridal pregnancies are associated with higher risks for PTB and LBW in Korea.

## Introduction

Social norms regarding marriage and family have changed dramatically. In Western countries, the link between marriage and childbirth has weakened over the past few decades. Studies from the U.S. have indicated that the proportion of women who bore children outside of marriage increased from 18.4% in 1980 to 41% in 2009 [1]. A significant increase in the number of infants born to unmarried women in European countries has also been noted [2], [3]. The recent increase in non-marital childbearing in Western countries has decreased the likelihood of marriage in response to pregnancy [4]. These trends might be caused by the increased social acceptance of non-marital childbirth and cohabitation in these countries [5].

In contrast, a strong relationship between marriage and childbearing still exists in East Asia. In Japan, 2% of all children are born to unmarried women, and the number of marriages in response to premarital conception (bridal pregnancy) has increased [4]. Nearly 18% of women are pregnant at the time of their marriage, and thus premarital pregnancy has become an increasingly important social issue. Given the increase in age at the first childbirth and the decrease in age in participation in sexual coitus, women are more likely to experience premarital conception. People in Asian countries still experience a strong social stigma associated with non-marital childbearing due to the influence of Confucian ideology and customs [6]. Thus, marriage in response to pregnancy is considered the normative pathway among societies in these countries [4].

Childbirth among unmarried women has been suggested to be associated with a higher risk of adverse pregnancy outcomes such as preterm birth (PTB), low birth weight (LBW), and small-for-gestational age infants [7]–[10]. Although the number of premarital pregnancies has increased for several decades in East Asia, no studies on the association between bridal pregnancy and obstetric outcomes among live births have been conducted to our knowledge. In this study, we used data collected from the Korean National Birth Registration (NBR) database to investigate the associations of bridal pregnancy on obstetric outcomes such as PTB and LBW, among live births.

## Materials and Methods

### Ethics statement

A statement from the local ethics committee was not necessary because no human experimentation was performed. The data used in our study were based on the Korean NBR database from National Statistical Office of Korea. In Korea, birth registration following childbirth is obligatory, and every parent must provide information on the maternal residential address at the time of birth, the date of birth, marital status, the date of marriage, gestational age, parental ages, parental education, parental occupations, sex, birth order, parity, and the total number of births. A birth certificate written by a physician or a nurse should be attached to the registration. The personal identification number used for all the data was deleted before being provided, and this study was thus performed using a secondary data analysis. For these reasons, this study was inapplicable to review from the ethical review panel [11].

### Study population

**Figure 1:**
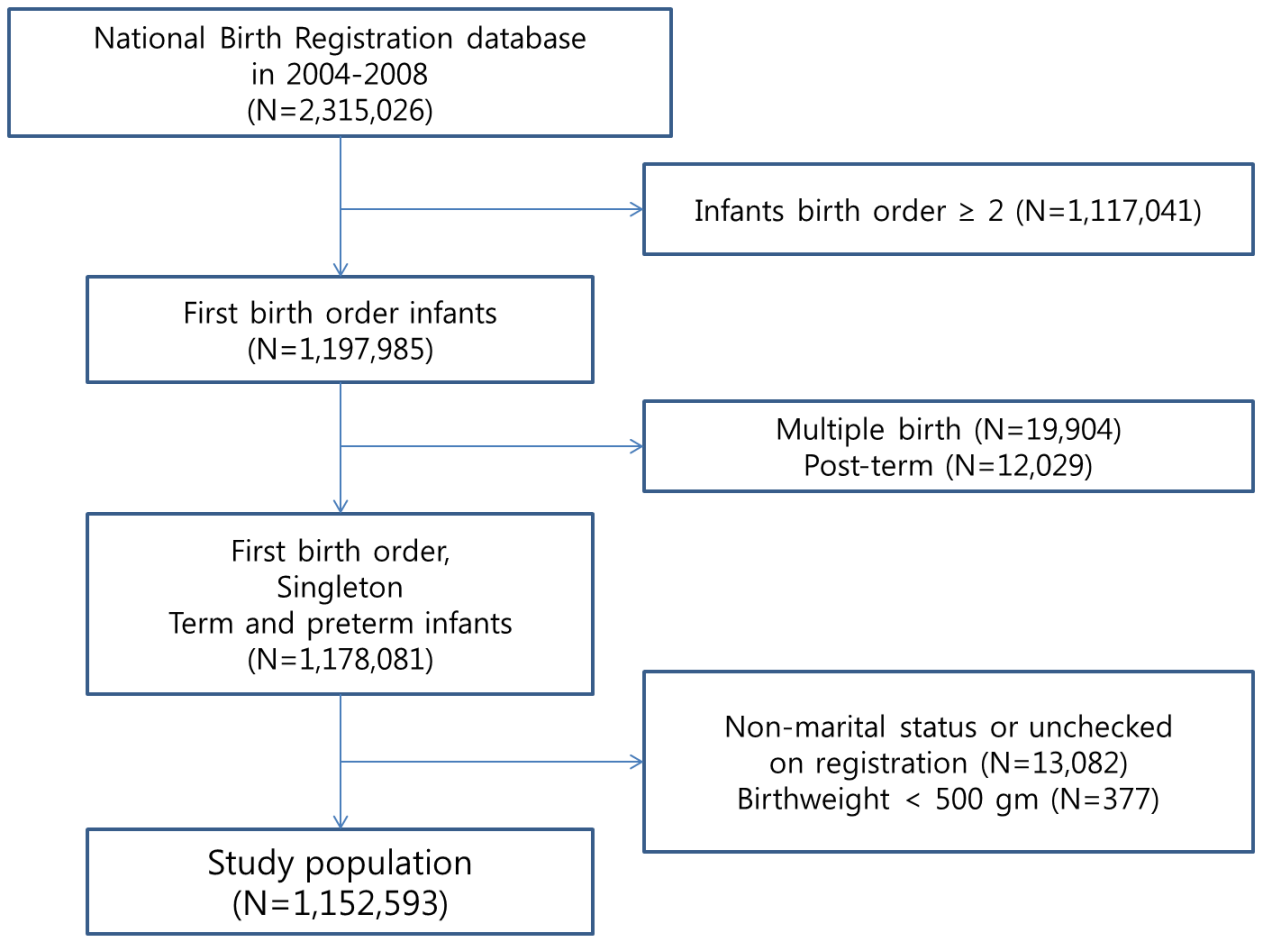
Flow diagrams depicting the selection of the study population.

Data on 2,315,026 births between 2004 and 2008 were obtained from the NBR database. Figure 1 depicts the model used to select our study population. We excluded second and greater birth order infants (N = 1,117,041) because most premarital pregnancies are related to first birth order infants. Multiple births and post-term pregnancies (greater than 42 completed weeks of gestation) were also excluded. Marital status was registered in approximately all cases, and those who were unmarried or had unchecked data were excluded (N = 13,082). Data from birth weights <500 g were also excluded because a birth weight of 500 g is in the zone where infant resuscitation is not clear. Finally, we selected 1,152,593 births for the study population.

**Figure 2:**
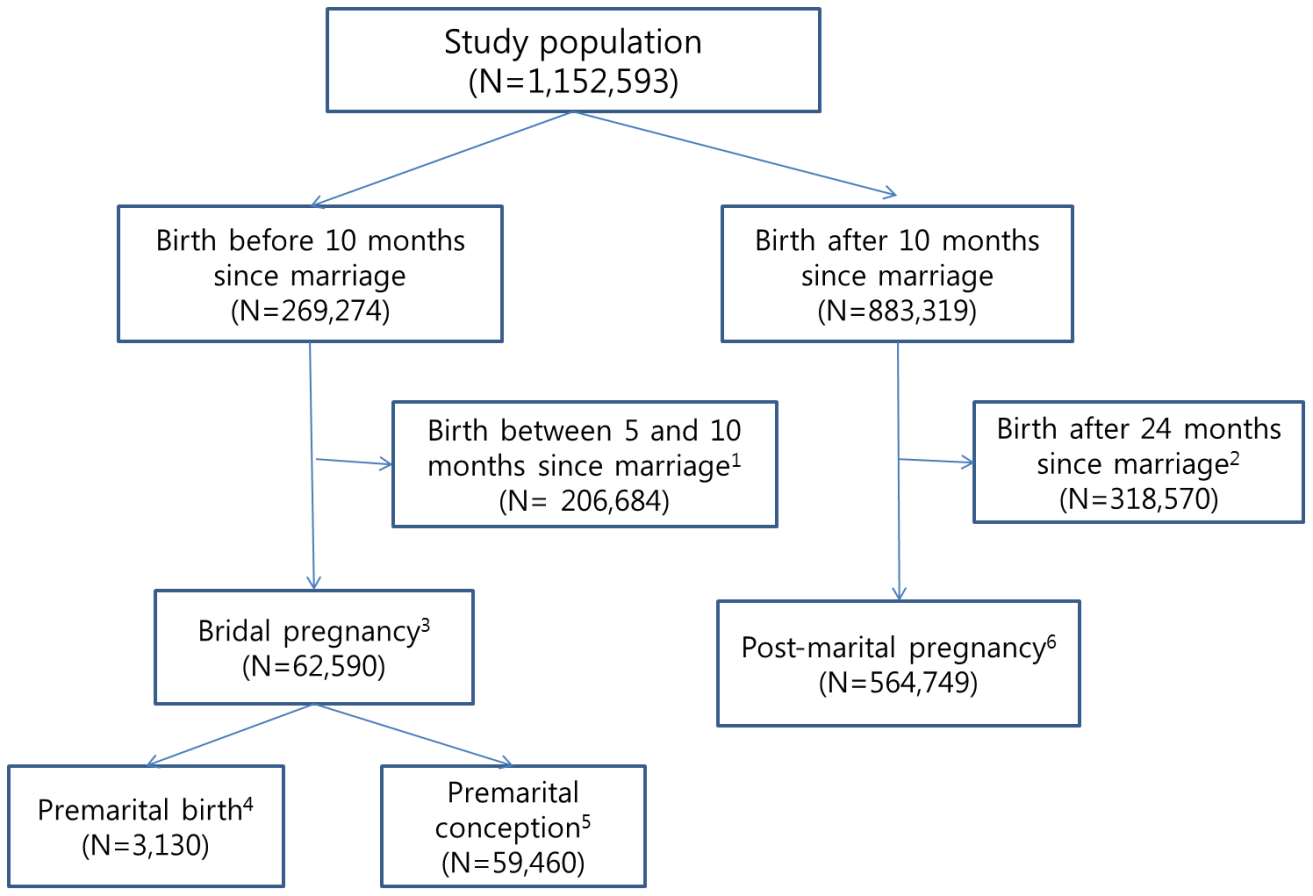
Flow diagrams illustrating the selection of the bridal pregnancy and post-marital pregnancy groups. ^1^ Because we could not distinguish premature births from post-marital pregnancy during this period, we excluded childbirths occurring between 5 and 10 months after marriage. ^2^ After 24 months of marriage, a certain portion of pregnancy outcomes may be affected by infertility treatment. Thus, we excluded births after 24 months of marriage. ^3^ Bridal pregnancy was defined as childbirth occurring before 5 months of marriage and was categorized as premarital birth and premarital conception. ^4^ Premarital birth was defined as childbirth before marriage followed by a marriage that occurred <1 month after birth. ^5^ Premarital conception was defined as pregnancy conceived before marriage and ending after marriage. ^6^ Post-marital pregnancy was defined as childbirth occurring between 10 and 24 months after marriage.

Figure 2 presents the process by which we chose the bridal pregnancy and post-marital pregnancy groups from the study population. We divided the study population into groups based on the timing of childbirth and marriage. Of the 1,152,593 individuals included, 269,274 infants were born before their parents had been married for 10 months and 883,319 infants were born after their parents had been married for at least 10 months. Bridal pregnancy (N = 62,590) was defined by excluding infants born between 5 and 10 months after marriage from those born before 10 months of marriage. Infants born between 5 and 10 months after marriage probably were most likely a mixed population of preterm births from the post-marital birth group and term births from the bridal pregnancy group. We could not identify whether premature births were conceived subsequent to marriage in that period, so we employed a very conservative definition of bridal pregnancy. Moreover, we divided bridal pregnancy into two categories, the premarital birth group and the premarital conception (but birth after marriage) group, based on the timing of childbirth. Premarital birth (N = 3,130) was defined as childbirth that occurred before marriage followed by a marriage that occurred after birth but before birth registration (<1 month from birth). Premarital conception (N = 59,460) was defined as a pregnancy conceived before marriage and ending after marriage.

To evaluate the obstetric outcomes among live births of bridal pregnancy, a reference group should be determined that has a minimal risk of adverse pregnancy outcomes. We defined the reference group as those women who gave birth after 10 months but before 24 months of marriage (N = 564,749) for two reasons. First, more than half of first childbirths occurred within 24 months of marriage (data not shown). Second, from this distribution, we inferred that a certain proportion of births would be affected by infertility treatment after 24 months, and therefore obstetric outcomes could be influenced by infertility treatments. Infants born from assisted reproductive technology are more likely to have adverse perinatal outcomes than spontaneously conceived infants [12], [13].

### Independent variables

Independent variables related to the socio-demographic status of the parents were also considered. These socio-demographic variables included birthplace, parental age, parental employment, and parental education level. Maternal and paternal ages were categorized as ≤19 years, 20–39 years, and ≥40 years. We categorized employment status as an office job, manual work, or unemployed. In the NBR database, subjects who were students and housewives were registered as unemployed, so we could not differentiate these individuals. Education was divided into 3 levels: below high school, high school, and college and higher.

### Types of outcome measures

PTB was defined as infants born before a gestational age of 37 weeks. Very preterm birth (VPTB) was defined as childbirth before a gestational age of 32 weeks. We defined LBW as infants weighing <2,500 g. Very low birth weight (VLBW) was defined as infants born weighing <1,500 g.

### Statistical Analysis

Descriptive statistics were reported for each response. Significant differences between the subjects and controls were evaluated by using χ^2^ tests for the categorical variables. Odds ratios (ORs) and 95% confidence intervals (CIs) were calculated by univariate analyses to evaluate the obstetric risks of bridal pregnancy. To identify associations between bridal pregnancy and adverse pregnancy outcomes, multiple logistic regression analyses were performed after controlling for the socio-demographic variables of birthplace, parental age, parental employment, and parental education. All tests were two-sided, and *P*-values <0.05 were considered significant. All data were analyzed using STATA 11.0 (StataCorp, College Station, Texas, USA).

## Results

### Demographic characteristics of the bridal pregnancy group compared with the post-marital pregnancy group

Table 1 presents the socio-demographic characteristics of the study population. All the demographic variables were significantly different between the 2 groups. Birthplace among the bridal pregnancy group was more likely to be in a rural area (7.99% vs. 6.53%, *P*<0.001), and the infants were more likely to be males (52.49% vs. 51.20%, *P*<0.001). In addition, compared with the post-marital pregnancy group more parents in the bridal pregnancy group were younger than 20 years (0.91% vs. 0.22% for paternal age (*P*<0.001), 2.29% vs. 1.13% for maternal age (*P*<0.001)). Furthermore, parents in the bridal pregnancy group were more likely to have a lower education level (*P*<0.001). Parental employment status also differed between the bridal pregnancy and reference groups (*P*<0.001).

**Table 1:** Socio-demographic characteristics of the bridal pregnancy and post-marital pregnancy groups.

	Bridal pregnancy	Post-marital pregnancy	*P*-value^1^
	(N = 62,590) %	(N = 564,749) %	
**Birth place**			
Urban	91.80	93.35	<0.001
Rural	7.99	6.53	
**Infant sex**			
Male	52.49	51.20	<0.001
Female	47.51	48.80	
**Paternal age**			
≤19	0.91	0.22	<0.001
20–39	96.73	96.07	
≥40	2.36	3.71	
**Maternal age**			
≤19	2.29	1.13	<0.001
20–39	97.18	98.30	
≥40	0.53	0.57	
**Paternal employment**			<0.001
Office	14.19	14.63	
Manual	74.43	78.54	
Unemployment	10.21	6.20	
Unknown	1.16	0.62	
**Maternal employment**			<0.001
Office	4.51	6.58	
Manual	13.43	23.20	
Unemployment	66.24	57.86	
Unknown	15.82	12.36	
**Paternal education**			<0.001
Below high school	3.34	2.68	
High school	45.60	30.40	
College or higher	50.85	66.83	
Unknown	0.21	0.09	
**Maternal education**			<0.001
Below high school	2.52	2.68	
High school	48.56	32.48	
College or higher	48.63	64.65	
Unknown	0.29	0.19	

1 Calculated using the χ^2^ test.

### Pregnancy outcomes among live births for the bridal pregnancy subgroups

We created the following bridal pregnancy subgroups: women who gave birth before marriage (premarital birth) and those who gave birth after marriage (premarital conception). Table 2 depicts the comparisons between the premarital birth and premarital conception subgroups, as well as the bridal pregnancy versus post-marital pregnancy groups. The multivariate analyses revealed increasing trends in the risks for PTB, VPTB, LBW, and VLBW among the bridal pregnancy, premarital conception, and premarital birth groups. Comparing the premarital birth group and the premarital conception group, increased risks for PTB (aOR: 1.19, 95% CI: 1.03–1.37) and VPTB (1.63, 1.30–2.53) were observed among the premarital birth group (not presented in the table).

**Table 2:** Pregnancy outcomes of the bridal (N = 62,590), premarital birth (N = 3,130), and premarital conception groups (N = 59,460) compared with post-marital pregnancy (N = 564,749).

	Group	%	*P*-value^1^	aOR^2^
**PTB**	Post-marital pregnancy^3^	3.4		1
	Bridal pregnancy^4^	5.97	<0.001	1.76 (1.69–1.82)
	- Premarital conception^5^	5.91	<0.001	1.74 (1.68–1.81)
	- Premarital birth^6^	7.12	<0.001	2.00 (1.74–2.29)
**VPTB**	Post-marital pregnancy^3^	0.62		1
	Bridal pregnancy^4^	1.57	<0.001	2.43 (2.26–2.62)
	- Premarital conception^5^	1.51	<0.001	2.36 (2.19–2.55)
	- Premarital birth^6^	2.74	<0.001	3.16 (2.89–4.52)
**LBW**	Post-marital pregnancy^3^	3.23		1
	Bridal pregnancy^4^	5.18	<0.001	1.53 (1.48–1.59)
	- Premarital conception^5^	5.18	<0.001	1.54 (1.48–1.59)
	- Premarital birth^6^	5.17	<0.001	1.48 (1.27–1.72)
**VLBW**	Post-marital pregnancy^3^	0.25		1
	Bridal pregnancy^4^	0.69	<0.001	2.77 (2.48–3.10)
	- Premarital conception^5^	0.69	<0.001	2.78 (2.48–3.11)
	- Premarital birth^6^	0.77	<0.001	2.61 (1.70–3.99)

PTB, preterm birth; VPTB, very preterm birth; LBW, low birth weight; VLBW, very low birth weight; aOR, adjusted odds ratio.

1 Calculated using the χ^2^ test.

2 Adjusted for birthplace, sex, paternal and maternal age, paternal and maternal employment, and paternal and maternal education level.

3 Post-marital pregnancy is defined as childbirth between 10 and 24 months of marriage.

4 Bridal pregnancy is defined as childbirth before 5 months of marriage. It is categorized according to the following subgroups : premarital conception and premarital birth.

5 Premarital conception is defined as pregnancy conceived before marriage and ending after marriage.

6 Premarital birth is defined as childbirth before marriage.

### Pregnancy outcomes among live births from bridal pregnancy according to maternal age

As presented in Table 3, univariate analyses revealed that bridal pregnancy was associated with an increased risk of PTB for a maternal age ≤19 years (OR 1.45, 95% CI: 1.13–1.84) or 20–39 years (1.82, 1.75–1.88) but not ≥40 years (1.16, 0.77–1.71) compared with the reference group. In the multivariate analysis, PTB was still significantly associated with bridal pregnancy among those with a maternal age ≤19 years (aOR 1.47, 95% CI: 1.15–1.89) or 20–39 years (1.76, 1.70–1.83). Bridal pregnancy was associated with a significantly increased risk for VPTB in the multivariate analysis for all maternal age groups compared with the reference group (aOR: 1.76 for ≤19 years, 2.42 for 20–39 years, and 1.90 for >40 years). However, in the categorical analysis of women with a maternal age ≤19 years or ≥40 years, the ORs for LBW and VLBW were not significantly different between the bridal pregnancy and reference groups in the univariate analyses. In the univariate analyses, bridal pregnancy was associated with an increased risk of LBW (OR: 1.66, 95% CI 1.59–1.72) and VLBW (2.86, 2.56–3.20) infants exclusively among those with a maternal age of 20–39 years. Moreover, the multivariate analyses revealed that infants born to the bridal pregnancy group were at a significantly higher risk of being LBW (aOR: 1.60, 95% CI: 1.53–1.66) and VLBW (2.70, 2.41–3.02) compared with those from the post-marital pregnancy group. However, this effect also was exclusively observed among women with a maternal age of 20–39 years.

**Table 3:** Pregnancy outcomes in bridal pregnancy and post-marital pregnancy, according to maternal age groups.

		Maternal age (≤19)			Maternal age (20–39)			Maternal age (≥40)		
		Bridal pregnancy^1^ (n = 1,433)	Post-marital pregnancy^2^ (n = 6,370)	*P*-value^3^	Bridal pregnancy^1^ (n = 60,825)	Post-marital pregnancy^2^ (n = 555,163)	*P*-value^3^	Bridal pregnancy^1^ (n = 332)	Post-marital pregnancy^2^ (n = 3,216)	*P*-value^3^
**PTB**	Crude proportion	6.69	4.72	0.002	5.93	3.36	<0.001	9.91	8.65	0.437
	Univariate OR	1.45 (1.13–1.84)	1		1.82 (1.75–1.88)	1		1.16 (0.77–1.71)	1	
	Multivariate OR^4^	1.47 (1.15–1.89)	1		1.76 (1.70–1.83)	1		1.13 (0.77–1.66)	1	
**VPTB**	Crude proportion	2.02	1.18	0.012	1.54	0.61	<0.001	4.5	2.48	0.029
	Univariate OR	1.73 (1.08–2.71)	1		2.58 (2.39–2.77)	1		1.86 (0.98–3.29)	1	
	Multivariate OR^4^	1.76 (1.11–2.79)	1		2.42 (2.25–2.61)	1		1.90 (1.06–3.39)	1	
**LBW**	Crude proportion	4.88	5.27	0.548	5.18	3.19	<0.001	6.91	6.1	0.563
	Univariate OR	0.92 (0.70–1.21)	1		1.66 (1.59–1.72)	1		1.14 (0.70–1.80)	1	
	Multivariate OR^4^	0.92 (0.70–1.21)	1		1.60 (1.53–1.66)	1		1.11 (0.71–1.74)	1	
**VLBW**	Crude proportion	0.7	0.38	0.096	0.69	0.24	<0.001	1.8	0.93	0.13
	Univariate OR	1.86 (0.79–4.04)	1		2.86 (2.56–3.20)	1		1.96 (0.66–4.82)	1	
	Multivariate OR^4^	1.79 (0.82–3.91)	1		2.70 (2.41–3.02)	1		1.99 (0.81–4.90)	1	

PTB, preterm birth; VPTB, very preterm birth; LBW, low birth weight; VLBW, very low birth weight; OR, odds ratio.

1 Bridal pregnancy is defined as childbirth before 5 months of marriage.

2 Post-marital pregnancy is defined as childbirth between 10 and 24 months of marriage.

3 Calculated using the χ^2^ test.

4 Adjusted for birthplace, sex, paternal age, paternal and maternal employment, and paternal and maternal education level.

## Discussion

To the best of our knowledge, this is the first study to demonstrate an association between bridal pregnancy and obstetric outcomes. We studied the associations of bridal pregnancy on pregnancy outcomes among live births, and found adverse pregnancy outcomes from marriage in response to pregnancy compared with the reference group. Subgroup analyses of women with a maternal age of 20–39 years indicated that the risk of adverse obstetric outcomes was greater in this age group compared with the other age groups (≤19 and ≥40 years). The elevated risks of PTB, VPTB, LBW, and VLBW in association with bridal pregnancy were significant. Moreover, the premarital conception group exhibited an increased risk for PTB and VPTB, and the greatest risk was observed in the premarital birth group.

Numerous previous studies have assessed maternal characteristics and health behaviors among unmarried women, demonstrating that unmarried mothers are generally younger, more often primiparous, more often unemployed, and smoke more than married women; however, only a few studies regarding bridal pregnancy have been reported [8], [14], [15]. Our study indicated that the characteristics of bridal pregnancy were significantly associated with a social disadvantage and particular risk factors for adverse pregnancy outcomes. However, this statistical significance should be interpreted with caution because the NBR database includes a very large sample size.

It would be worthwhile to classify the study population according to age because an extreme age group is itself a risk for adverse obstetric outcomes. As a result, the associations of bridal pregnancy on pregnancy outcomes differed based on the maternal age group. Women ≤19 and ≥40 years of age were in the extreme age groups, and studies have demonstrated that infants from these 2 extreme maternal age groups already exhibited an increased risk for adverse obstetric outcomes compared with women with a maternal age of 20–39 years, and thus differences in the pregnancy outcomes from bridal pregnancy were not prominent in these groups [16]–[18].

Pregnancy outside of marriage is a well-known risk factor for PTB, LBW, and for small–for-gestational age infants [10], [14], [19]. Higher aORs for PTB and VPTB were observed in the premarital birth group compared with the premarital conception group. These results are consistent with those from Finland [10].

We observed higher ORs for adverse obstetric outcomes among the bridal pregnancy group after controlling for socio-demographic factors. To date, little information is known regarding the mechanisms associated with adverse outcomes in premarital birth. We suggest several possible mechanisms by which bridal pregnancy can be associated with adverse pregnancy outcomes. First, almost every conception that can be defined as a bridal pregnancy would be an unintended pregnancy. Previous studies have shown that unintended pregnancies are significantly associated with higher risks for PTB and LBW [20], [21]. Second, prenatal stress might be higher among women who experience bridal pregnancy compared with post-marital pregnancy. Pregnant women who have higher stress levels exhibit a higher risk of preterm delivery and stillbirth [22]–[25]. Higher stress is expected among women in the bridal pregnancy group because they are likely aware of social stigma. In addition, these women are preparing for a wedding, which is also stressful for couples. Third, poor behavioral factors, such as smoking, alcohol, obesity, and multiple sexual partners, could be significantly associated with bridal pregnancy.

A limitation of our study is that the independent variables including marital status were primarily based on self-reported registration data. The birth registration database also does not contain sufficient information on potential confounders of maternal factors, such as obesity, smoking, alcohol, and sexual behaviors, which are already known to be risk factors of adverse pregnancy outcomes [26], [27]. Information associated with obstetric outcomes, such as stress level, completion of prenatal checks, and chronic illness, could not be assessed. We also could not estimate factors mediating adverse pregnancy outcomes with bridal pregnancy. In addition, no information regarding the method used to estimate gestational age in the NBR database was available. The last menstrual period has been used to estimate gestational age, but this metric may not be equal to the real gestational age if the menstrual period is irregular. Third, we only had limited data on the socioeconomic status of the study population given the education and employment classifications used. Students and housewives were classified as unemployed and students were classified according to current educational status. Lastly, we could not include fetal loss rate including stillbirths and induced abortion cases in the analysis because only live births are registered in the NBR database.

In this nationally representative survey of the Korean NBR database, bridal pregnancy was associated with a higher risk for PTB and LBW. For bridal pregnancy to be reflective of other social, environmental, or behavioral conditions that were not included in the present study, further studies considering these variables are required.
